# Association of Prenatal and Intrapartum Antibiotic Use with Risk of Childhood Atopic Dermatitis: A Systematic Review and Meta-Analysis

**DOI:** 10.3390/children12070859

**Published:** 2025-06-30

**Authors:** Yu-Chuan Chang, Hsing-Ju Wu, Meng-Che Wu

**Affiliations:** 1 Department of Pediatrics, Chang Bing Show Chwan Memorial Hospital, Changhua 505, Taiwan; 2 Research Assistant Center, Show Chwan Memorial Hospital, Changhua 500, Taiwan; 3 Department of Medical Research, Chang Bing Show Chwan Memorial Hospital, Changhua 505, Taiwan; 4 Department of Nursing, Jenteh Junior College of Medicine, Nursing and Management, Miaoli 356, Taiwan; 5 Division of Pediatric Gastroenterology, Children’s Medical Center, Taichung Veterans General Hospital, Taichung 407, Taiwan; 6 School of Medicine, Chung Shan Medical University, Taichung 402, Taiwan; 7 Department of Post-Baccalaureate Medicine, College of Medicine, National Chung Hsing University, Taichung 402, Taiwan

**Keywords:** prenatal antibiotics, atopic dermatitis, intrapartum antibiotic prophylaxis, prenatal exposure, eczema, pregnancy

## Abstract

**Background/Objectives**: Atopic dermatitis (AD) is a chronic inflammatory skin condition with rising global prevalence. Increasing maternal antibiotic use during pregnancy has raised concerns about its potential link to childhood allergic diseases, including AD. However, existing meta-analyses have yielded inconsistent results. A systematic review and meta-analysis were conducted to investigate the association between prenatal antibiotic exposure, including intrapartum antibiotic prophylaxis (IAP), and the risk of AD developing in offspring. **Methods**: A systematic search protocol (PROSPERO ID: CRD42024577804) was conducted up to 29 August 2024, across the PubMed, Embase, and Cochrane databases. Cohort and case–control studies reporting associations between maternal antibiotic exposure during pregnancy or intrapartum and the risk of AD in offspring were included. Data were analyzed using RevMan Web and Comprehensive Meta-Analysis software. **Results**: Twenty studies involving 3,256,929 mother–child pairs were reviewed. The meta-analysis data demonstrated that prenatal antibiotic exposure was associated with AD in the main analysis (odds ratio [OR]: 1.12, 95% CI 1.03–1.21), but not in a separate analysis with a pooled hazard ratio (HR) (HR: 1.12, 95% CI 0.96–1.31). Trim-and-fill correction for significant publication bias (Egger’s test *p* = 0.003) in the main analysis resulted in a non-significant effect size (OR: 1.09, 95% CI 0.99–1.20). Subgroup analysis and meta-regression suggested that publication years and sample sizes contributed significant heterogeneity (*p* < 0.05). Regarding IAP and the risk of AD, no association was found (OR: 1.62, 95% CI 0.87–3.00). **Conclusions**: Current evidence in the existing literature does not support a positive relationship between antibiotic exposure, either during pregnancy or in the intrapartum period, and the risk of development of AD in offspring. However, substantial heterogeneity and the very low certainty of evidence limit the strength of our findings. Further studies that address confounders more thoroughly are needed to confirm these results.

## 1. Introduction

Atopic dermatitis (AD) is a chronic inflammatory skin disease characterized by pruritus, lichenification, and xerosis [1]. Despite a decline in the age-standardized prevalence of AD from 1990 to 2019, its total prevalence, incidence, and associated disability-adjusted life years have increased, leading to substantial morbidity [2]. The etiology of AD is likely multifactorial, involving genetic predisposition, immune dysfunction, and environmental and lifestyle factors [3]. Th2-skewed inflammation, characterized by elevated IL-4, IL-13, and IL-31, is a hallmark of AD and contributes to skin inflammation and pruritus [4]. Filaggrin (FLG) gene mutations and skin or gut microbial dysbiosis further impair barrier function and immune tolerance, promoting disease development [4]. Regions with a higher socio-demographic index report a higher incidence of AD [2], which can likely be explained by the “hygiene hypothesis”, which posits that reduced microbial exposure in early life may disrupt immune system development and contribute to atopy.

Approximately a quarter of women worldwide use antibiotics during pregnancy, with a meta-analysis estimating the pooled prevalence at 23.6% (95% CI 20.1–27.5) [5]. A study from Germany found urinary tract infections to be the most common reason for prescribing antibiotics, followed by respiratory infections [6]. Additionally, intrapartum antibiotic prophylaxis (IAP), primarily used for preventing Group B *Streptococcus* (GBS) disease, accounts for a portion of antibiotic use during pregnancy [7]. The global maternal GBS colonization rate is estimated to be approximately 18%, with regional variations, and a North American study found that approximately two-thirds of colonized pregnant women received antibiotic prophylaxis during the intrapartum period [8,9]. Hypothesized mechanisms suggest that the maternal microbiome may influence the maturation of fetal epithelium and immune cells through cytokines, hormones, or bacterial products during pregnancy, as well as through changes in neonatal gut microbiomes during childbirth [10,11]. Dysbiosis induced by maternal antibiotic exposure could lead to subsequent allergic disease in childhood. This was demonstrated in a mouse model showing mother-to-offspring transfer of antibiotic-induced gut microbial dysbiosis, affecting the offspring’s immune system [12].

Although some meta-analyses have investigated the association between maternal antibiotic exposure and AD risk, the effect estimates have been inconsistent [13,14,15,16,17]. A possible explanation for the inconsistency may be that some results were based on unadjusted data or the combination of different trimesters in the analyses. Additionally, these analyses either included a limited number of studies (fewer than 10) or showed very high heterogeneity (*I*^2^ > 75%), weakening the robustness of the results [13,14,15,16,17]. Notably, no prior meta-analysis has specifically examined the relationship between IAP and AD risk. To address these issues, we performed a systematic review and meta-analysis to investigate the association of prenatal antibiotic and IAP exposure with the risk of AD, incorporating updated studies from the past three years, using adjusted pooled results, and trying to elucidate possible confounders.

## 2. Materials and Methods

This meta-analysis adhered to the guidelines of the Preferred Reporting Items for Systematic Reviews and Meta-Analyses (PRISMA) statement (Table S1) [18]. The protocol was registered with the International Prospective Register of Systematic Reviews (PROSPERO) prior to data extraction under the following registration ID: CRD42024577804.

### 2.1. Eligibility Criteria

Studies were included if they met the following criteria: (a) original articles using cohort, case–control, or cross-sectional designs; (b) maternal antibiotic exposure during pregnancy or during the intrapartum period as the exposure of interest; (c) the development of AD in offspring as the outcome; and (d) effect sizes reported as odds ratios (ORs), hazard ratios (HRs), or risk ratios (RRs) with 95% confidence intervals (CIs), or sufficient data to calculate them.

Studies were excluded if they (a) reported only unadjusted effect sizes and CI; (b) used comparison groups that were not “no antibiotic exposure” during the relevant exposure period (e.g., comparing pre- vs. post-incision antibiotic exposure or high vs. low doses of antibiotics); (c) reported only combined allergic outcomes, instead of AD-specific outcomes; and (d) were conference abstracts or reviews.

Additionally, we obtained type-specific antibiotic exposure information from one study [19], data on antibiotic administration via the oral route from another [20], and confirmation of adjusted analyses from a third study [21] after consulting with the authors.

### 2.2. Literature Search

A comprehensive literature search was conducted, up to 29 August 2024, in PubMed, Embase, and the Cochrane Central Register of Controlled Trials (CENTRAL), with no specific language restrictions. The search strategy is detailed in Table S2.

### 2.3. Article Selection and Data Extraction

**Figure 1:**
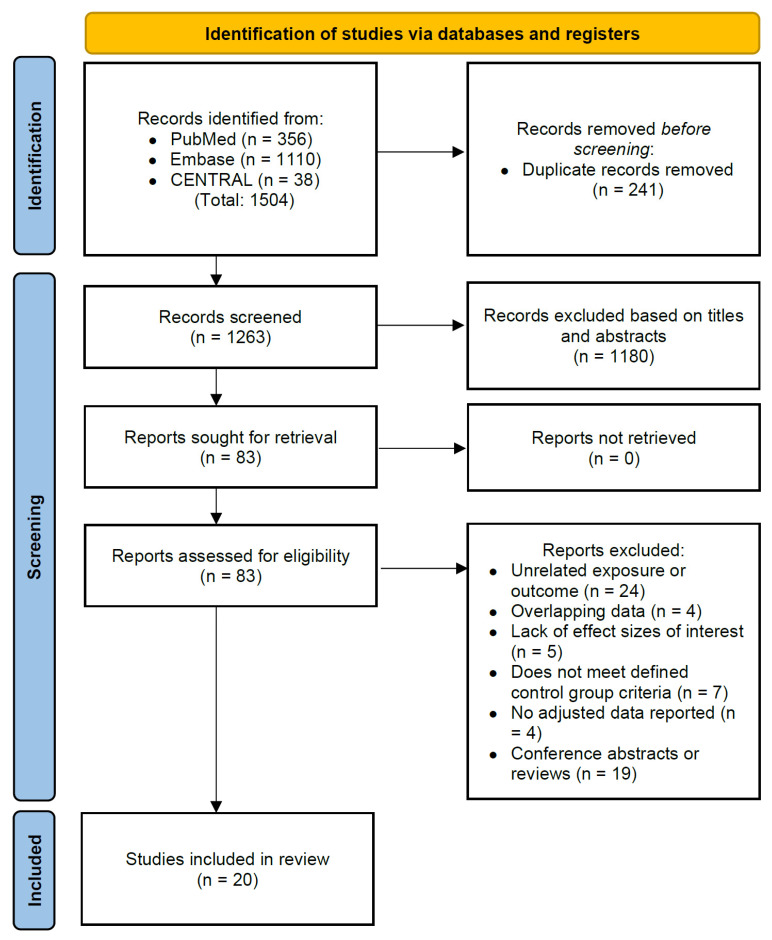
PRISMA flow diagram of article selection for the systematic review.

All retrieved records from the search were imported into Endnote X9 (Clarivate Analytics) for deduplication. Two independent reviewers (Y-C Chang and H-J Wu) performed further deduplication, screened titles and abstracts, and assessed full-text eligibility. Study selection followed PRISMA guidelines, and the selection process is detailed in the PRISMA flow diagram (Figure 1). Studies that did not meet the eligibility criteria were excluded. The following data were extracted: the first author’s last name, year of publication, study design and population, sample size, age at diagnosis, methods of exposure and outcome measurements, effect sizes, and adjusted confounders. In addition, data on effect sizes for specific trimesters of exposure, types of antibiotics, and the number of courses were also extracted if the relevant data were provided. For overlapping data, only the study with the largest sample size and most complete confounder adjustment was included. Disagreements were resolved by consensus or by involving a third reviewer (M-C Wu).

### 2.4. Quality Assessment

The risk of bias and methodological quality of the included studies were assessed using the Newcastle–Ottawa Scale (NOS), which is widely used for cohort and case–control studies [22]. It consists of three categories: selection, comparability, and outcome (for cohort studies) or exposure (for case–control studies). The scoring allows a maximum of 4 stars in selection, 2 stars in comparability, and 3 stars in outcome/exposure. Two reviewers (Y-C Chang and H-J Wu) conducted assessments, resolving disagreements by consensus. Additionally, subgroup analyses were performed based on study quality to examine whether effect estimates varied by risk of bias.

### 2.5. Certainty Assessment

The Grading of Recommendations, Assessment, Development, and Evaluations (GRADE) approach was used to determine the certainty of the evidence, considering study limitations, inconsistency, indirectness, imprecision, and publication bias. Evidence quality was rated as high, moderate, low, and very low. Initially, observational studies were rated at a low level, but could be either upgraded or downgraded based on further evaluation [23,24]. Two independent reviewers (Y-C Chang and H-J Wu) conducted the certainty assessment. Any disagreements were first discussed between the two reviewers. If consensus was not reached, a third reviewer (M-C Wu) was consulted for arbitration.

### 2.6. Statistical Analysis

#### 2.6.1. Data Preparation and Synthesis

The adjusted OR or HR and its 95% CI was used as the association measure for all studies. The OR and HR were pooled separately, since they convey different meanings and are not directly comparable [25]. In studies that did not report a composite outcome of associations but reported outcomes for different subgroups, we pooled them together using meta-analysis software. However, for the analysis of IAP and its association with AD risk, we pooled the data by subgroups according to the mode of delivery, as the study by Wohl et al. included only participants from vaginal deliveries [21].

#### 2.6.2. Publication Bias and Sensitivity Analysis

Publication was tested by visually inspecting the funnel plot asymmetry and Egger’s test of the intercept. If publication bias was detected (*p* < 0.05, 2-tailed), Duval and Tweedie’s trim-and-fill method was applied to recalculate the effect sizes. Sensitivity analysis was performed using the “one study removal” method to detect outliers. Subgroup analyses were conducted based on multiple factors, including study type, design, region, sample size, publication year, exposure and outcome assessment, adjusted confounders, NOS, trimesters of exposure, types of antibiotics, and number of courses. These factors were also included in univariate meta-regression as moderators to better understand heterogeneity and predictors of outcomes. Directed acyclic graphs (DAGs) were constructed to identify potential confounders (Figure S1).

#### 2.6.3. Model Estimation

Both the meta-analysis and meta-regression used random-effects models, since the included studies represented samples from different populations with varying demographics [26]. Heterogeneity was evaluated through Cochran’s Q test and *I*^2^ statistic, with *p* < 0.05 considered significant and *I*^2^ > 50% indicating substantial heterogeneity [27,28]. The main analyses were performed using the Review Manager software (RevMan Web, The Cochrane Collaboration, London, UK), whereas publication bias and meta-regression were evaluated by Comprehensive Meta-Analysis software (CMA, Version 4.0, Biostat Inc., Englewood, NJ, USA).

## 3. Results

### 3.1. Study Selection, Characteristics, and Quality Assessment

A total of 1504 articles were initially identified through a systematic search. After excluding 241 duplicate articles and 1180 articles based on title and abstract review, 63 additional articles were found to be ineligible, resulting in 20 articles being included in the meta-analysis (Figure 1).

The included studies had publication years ranging from 2002 to 2023, and comprised 18 cohort studies [19,20,21,29,30,31,32,33,34,35,36,37,38,39,40,41,42,43] and 2 case–control studies [44,45]. The studies collectively included 3,256,929 mother–child pairs and were conducted in Europe (*n* = 12), Asia (*n* = 5), and North America (*n* = 3). Sixteen studies examined the association between maternal antibiotic exposure during pregnancy and the risk of AD [19,20,29,31,32,33,35,36,37,38,39,40,41,42,43,44], while four studies focused on IAP and AD risk [21,30,34,45]. Only a few studies explored specific factors such as the types [19,29], timing [29,40], and number of antibiotic courses [29,40] in relation to AD risk. Additionally, there was considerable heterogeneity in the confounders adjusted across the studies. All the detailed study characteristics are presented in Table 1.

The NOS assessment (Table 2) indicated that most studies (80%) were of high quality, with scores ranging from 7 to 9.

### 3.2. Association of Prenatal Antibiotic Exposure with Risk of AD

**Figure 2:**
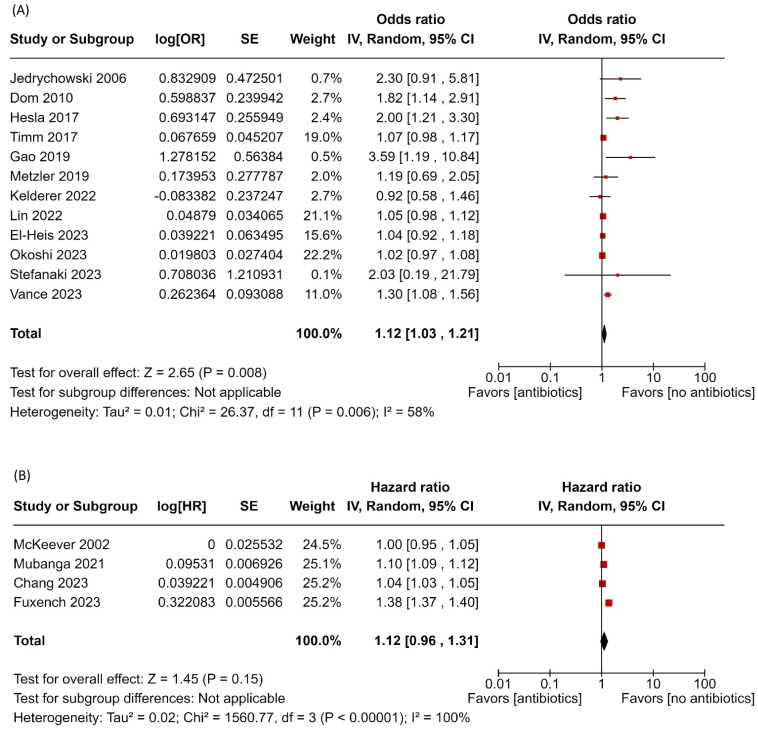
Forest plot of prenatal antibiotic exposure and risk of atopic dermatitis. (**A**) Main analysis with pooled estimates from 12 studies reporting ORs; (**B**) separate analysis pooling estimates from studies that reported HRs [19,20,29,31,32,33,35,36,37,38,39,40,41,42,43,44]. Red squares represent individual study estimates, and black diamonds indicate the pooled effects.

Sixteen studies examined the relationship between maternal antibiotic use during pregnancy and the risk of childhood AD. Among these, twelve studies reported effect sizes as ORs, while four used HRs. The pooled OR from the twelve studies indicates that prenatal antibiotic exposure is associated with an increased risk of childhood AD (OR: 1.12, 95% CI: 1.03–1.21; *I*^2^ = 58%, *p* = 0.006) (Figure 2). However, the effect estimates from the four studies reporting HRs did not reach statistical significance (HR: 1.12, 95% CI: 0.96–1.31; *I*^2^ = 100%, *p* < 0.00001) (Figure 2). Sensitivity analysis of the twelve studies showed consistency among the results (Figure S2). In contrast, the effect estimates from the separate four studies showed a significant increase after excluding the study by Fuxench et al. (Figure S3).

**Figure 3:**
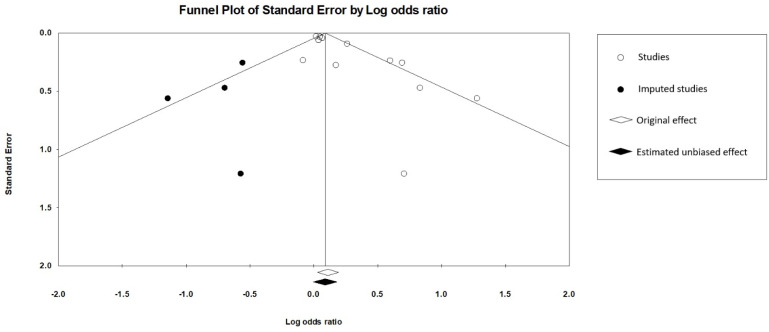
Funnel plot of main meta-analysis including 12 studies and 4 imputed studies.

For the main meta-analysis of the twelve studies, visual inspection of the funnel plot indicated asymmetry (Figure S4), and an Egger’s test revealed significant publication bias (intercept = 1.58, 2-tailed *p* value = 0.003). The trim-and-fill method was performed to correct this bias, which resulted in a non-significant effect (OR: 1.09, 95% CI 0.99–1.20), suggesting that publication bias may have inflated the effect (Figure 3).

### 3.3. Association of IAP with Risk of AD

**Figure 4:**
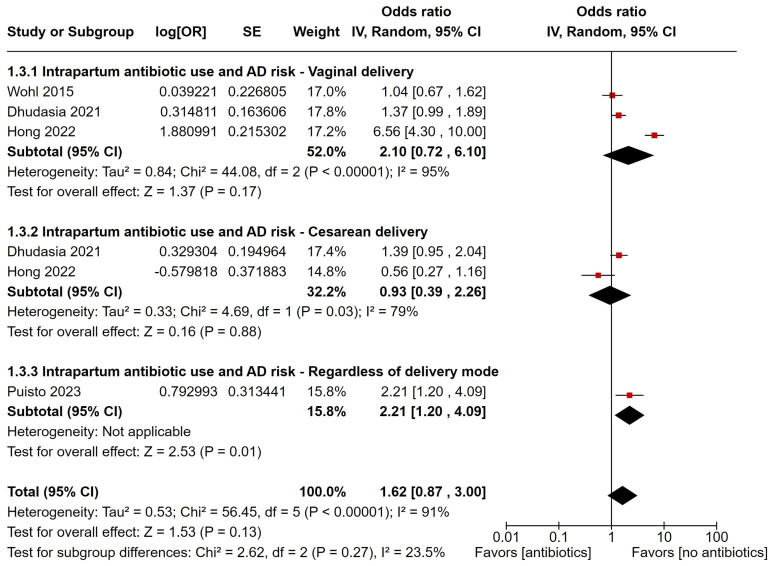
Forest plot of intrapartum antibiotic prophylaxis and risk of developing atopic dermatitis, with estimates grouped by mode of delivery [21,30,34,45]. Red squares represent individual study estimates, and black diamonds indicate the pooled effects.

Four studies investigated the link between IAP and AD. Of these, three reported ORs for vaginal delivery, two for cesarean delivery, and one for a composite outcome. The pooled estimates showed that IAP did not significantly increase AD risk (OR: 1.62, 95% CI 0.87–3.00), though the risk was higher for vaginal deliveries (OR: 2.10, 95% CI 0.72–6.10) compared to cesarean deliveries (OR: 0.93, 95% CI 0.39–2.26) (Figure 4). There was high heterogeneity among the data (*I*^2^ = 91%, *p* < 0.00001). Sensitivity analysis showed that removing the study by Hong et al. significantly increased the AD risk associated with IAP (Figure S5). Due to the small number of studies, Egger’s test was not interpretable, though publication bias could not be ruled out based on the funnel plot.

### 3.4. Subgroup Analysis and Meta-Regression

Subgroup analysis (Table 3) identified several factors associated with a decreased risk of AD related to prenatal antibiotic use. Specifically, studies conducted in Asia, those with larger sample sizes, more recent publications, non-parental or interview-based assessments of exposure and outcomes, and those with an NOS score ≥ 8 were linked to a reduced risk. Notably, studies that adjusted for maternal infections or paracetamol use, as well as child infections or antibiotic use, showed a non-significant association. Conversely, studies that adjusted for parental allergic diseases, first-trimester exposure, use of macrolide antibiotics, and having five or more antibiotic courses were associated with a slightly increased risk of AD. In the meta-regression model, significant moderators included publication years and sample sizes (*p* < 0.05) (Table 3).

### 3.5. Grading of Evidence

According to the GRADE approach, the effect of prenatal antibiotic use on the risk of AD was rated as very low, both for effect estimates expressed as ORs and HRs. Similarly, the effect of IAP on the risk of AD was also rated as very low. Reasons for this grading included concerns about inconsistency, publication bias, and imprecision, as detailed in Table 4. While a dose–response relationship was observed in the HR group, it did not influence the overall grading score.

## 4. Discussion

### 4.1. Main Findings

The goal of this study was to provide an updated systematic review and meta-analysis examining the associations of maternal antibiotic exposure during pregnancy and the intrapartum period with AD outcomes. To our knowledge, no prior meta-analysis has specifically investigated the effect of IAP on AD. The main analysis showed a positive association between prenatal antibiotic use and AD, which is consistent with previous studies. This could be due to maternal microbiome disruption, which affects fetal immunity and plays a crucial role in the development of the fetal immune system [11]. However, this primary finding may be influenced by publication bias, as the effect size became non-significant after applying the trim-and-fill method. Additionally, a separate analysis that pooled four studies reporting HRs found no association. These findings suggest that prenatal antibiotic use may not be associated with AD as previously reported.

Regarding IAP, our analysis found no association with the risk of AD. Previous meta-analyses have suggested that maternal IAP is associated with a non-significant reduction in infant microbiome diversity, an effect that seems to be temporary [46]. This transient impact might explain the lack of significant association in our study. Additionally, the higher association observed in the vaginal delivery group compared to the cesarean delivery group could be attributed to the greater impact of reduced microbial diversity in vaginal deliveries. However, research on IAP and AD is limited, as our analysis included only four studies.

### 4.2. Additional Findings

Several confounders, as depicted in the DAG, may influence the association between prenatal antibiotic use and AD. Maternal infections, shared environmental factors, postnatal antibiotic use, and healthcare-seeking behaviors could confound the relationship between exposure and outcome, contributing to both “confounding by indication” and “protopathic bias” [47]. Our subgroup analyses suggested that studies adjusting for these factors showed a weaker effect than those that did not, although this difference was not significant in the meta-regression model (*p* = 0.82). The inconsistency in adjusted confounders between studies and the limited study numbers might explain the non-significance, but further research is needed to fully address these variables.

Our meta-regression identified a “decline effect”, where the strength of associations reported in studies diminished over time [48]. This phenomenon may be attributed to factors such as selective reporting, publication bias, and small sample sizes [48]. This was further supported by our analysis, which showed that studies with larger sample sizes reported less significant effects. Regarding heterogeneity, our analysis also showed that subgroups based on sample sizes and publication years demonstrated more homogeneous results. Another subgroup with less heterogeneity included studies that reported exposure and outcome measurements based on database or medical records, suggesting that the use of objective, standardized data sources likely reduced variability in the reported effect sizes; however, this subgroup only included two studies, and therefore should be interpreted cautiously. Regarding IAP, a dose–response relationship was identified in the study by Wohl et al., where IAP administration for more than 24 h was associated with a higher risk of AD [21]. However, this effect was not observed in the study by Dhudasia et al. [30]. Subgroup analysis could not be performed due to incomplete statistical data.

In the sensitivity analysis, the main analysis demonstrated robust and consistent results. However, we observed that removing the studies by Fuxench et al. [19] and Hong et al. [34] shifted the risk estimates from non-significant to significant in the separate analyses of HRs and the association of IAP with AD risk, respectively. These two studies reported more pronounced effect estimates compared to the others. Possible explanations may be as follows. First, there were residual confounders, such as maternal infections during pregnancy, healthcare-seeking behaviors (as previously mentioned), the mode of delivery, and child characteristics that were not adjusted for in the study by Fuxench et al., which may have inflated the outcome estimates. Second, the cesarean delivery rate in the study by Hong et al. (48%) was higher than that in the general population, possibly leading to an overrepresentation of high AD risk in the vaginal delivery group. Third, demographic or genetic differences between the studies cannot be ruled out; for example, the GBS colonization rate in the study by Hong et al. (12.6%) was lower than the global average [8]. These factors may have influenced the sample representation and contributed to the shift in effect observed in the sensitivity analysis.

### 4.3. Comparison with Existing Meta-Analyses

The first meta-analysis regarding the effect of prenatal antibiotic exposure on AD was conducted in 2013 by Tsakok et al. [15], which found no association. However, subsequent meta-analyses reported positive associations [13,14,16,17]. Compared to their studies, our meta-analysis, which more appropriately analyzed ORs and HRs separately, demonstrated reduced heterogeneity and systematically addressed publication bias. We included seven studies published more recently, since 2021, that were previously not included [19,29,30,32,41,43,44]. The larger sample sizes and the inclusion of more controlled variables in these newly published studies likely contributed to the reduction in heterogeneity in our analysis. The greater number of studies enhances the robustness and reliability of our findings.

### 4.4. Strengths and Limitations

Strengths of this systematic review and meta-analysis include the use of comprehensive search strategies, ensuring that all relevant studies were included. We addressed publication bias statistically with the trim-and-fill method and performed meta-regression to identify potential effect modifiers. Additionally, the use of DAGs and subgroup analyses allowed for the visualization and assessment of confounding structures. The GRADE framework also enhanced transparency and reliability.

However, several limitations should be noted: (a) The number of studies examining IAP exposure and the risk of AD was relatively small (*n* = 4), which may have limited the power and generalizability of the findings. Similarly, some subgroups also had few studies, restricting the exploration of heterogeneity and robust meta-regression. (b) The included studies adjusted for inconsistent sets of confounders, with most unable to address “confounding by indication” or “protopathic bias”. This inconsistency may have contributed to the heterogeneity observed in this meta-analysis. (c) We did not search for gray literature, which may have led to the omission of some studies. (d) As the GRADE assessment suggests, the certainty of the included studies was categorized as “very low”, thereby limiting the study results.

## 5. Conclusions

Given the widespread use of antibiotics during pregnancy, understanding their potential long-term effects on offspring health is critical. While the primary analysis showed a positive association between prenatal antibiotic exposure and the risk of AD, this association disappeared after publication bias adjustment. Additionally, no association was found in separate analyses using pooled HR data or examining IAP exposure and the risk of AD.

Overall, current evidence does not support a consistent positive association between prenatal antibiotic use and AD in offspring. These findings may help to inform clinical decision-making regarding antibiotic use during pregnancy and childbirth. Future research should prioritize comprehensive adjustments for confounding factors, objective assessments of exposure and outcomes, and dose–response analyses (e.g., prolonged IAP use and its link to AD).

## Figures and Tables

**Table 1:** Characteristics of included studies.

Author, Year	Study Design, Country	Total Numbers	Age	Exposure Measurement	Outcome Measurement	Outcome Measures	Adjusted Confounders
Mckeever, [38] 2002	Retrospective cohort, United Kingdom	24,690	0–11	Database records (medical prescriptions)	Database records (doctor diagnoses)	Pooled HR: 1.00, 95% CI: 0.95–1.05	Consulting behavior in first 6 months, maternal atopy, and maternal infections in pregnancy
Jedrychowski, [35] 2006	Prospective birth cohort, Poland	102	0–1	Parent reports through interviews	Parent reports through interviews	aOR: 2.3, 95% CI: 0.91–5.8	Maternal education, child’s gender, maternal allergy, and number of child respiratory infections
Dom, [31] 2010	Prospective birth cohort, Belgium	773	0–4	Parent reports during home visits	Parent reports through questionnaires	aOR: 1.82, 95% CI: 1.14–2.92	Gender, maternal age, birth weight, siblings, breastfeeding, pre- and postnatal exposure to cats or dogs, pre- and postnatal exposure to cigarette smoke, day care attendance, parental education and parental history of allergies, and postnatal antibiotics
Wohl, [21] 2015	Retrospective cohort, United States	492	0–2	Medical records	Medical diagnosis or prescription	Estimated aOR: 1.04, 95% CI: 0.67–1.63	Sex, birth order, parental eczema/asthma, sibling eczema, pet ownership, GBS status, smoking, infant feeding during first 3 months, gestational age, birth weight, and maternal education
Hesla, [37] 2017	Prospective birth cohort, Sweden	490	0–2	Parent reports	Parent reports through questionnaires through interview	aOR: 2.0, 95% CI: 1.2–3.3	Lifestyle factors, residence type (apartment), farm exposure (living on a farm with animals), recent indoor painting (child’s room), household pet ownership, and time for first wash of whole body
Timm, [42] 2017	Prospective birth cohort, Denmark	62,560	0–1.5	Parent reports through telephone interviews	Parent reports through telephone interviews	Pooled aOR: 1.07, 95% CI: 0.98–1.17	Prenatal smoking, household socio-economic status, and older siblings
Gao, [33] 2019	Prospective birth cohort, China	903	0–1	Parent reports through questionnaires	Parent reports of doctor-diagnosed eczema through questionnaires during home visits	aOR: 3.59, 95% CI: 1.19, 10.85	Infant’s sex, maternal consumption during pregnancy, parental allergy, mode of delivery, postpartum depression, season of birth, siblings’ numbers, breastfeeding, age of solid food introduction, and antibiotic exposure in first year of life
Metzler, [39] 2019	Prospective birth cohort; Austria, Finland, France, Germany, and Switzerland	1080	0–6	Parent reports through questionnaires via interview	Parent reports of doctor’s diagnosis through questionnaires	aOR: 1.19, 95% CI: 0.69–2.05	Farming status, study center, parental atopic status, gender, smoking during pregnancy, number of siblings, pets (dogs and cats) exposure during pregnancy, cesarean section, maternal education
Mubanga, [40] 2021	Prospective cohort study, Sweden	722,767	5.8 ± 2.4 (mean ± SD)	Database records (dispensed prescription)	Database records (AD registration + medical prescription)	aHR: 1.10, 95% CI: 1.09–1.12	Age, sex, mother’s age, family situation, parity, level of education, area of residence, smoking history, maternal history of asthma, and mode of delivery
Dhudasia, [30] 2021	Retrospective cohort study, United States	14,046	0–5	Medical records	Medical records (diagnosis codes + prescription of topical steroids)	Vaginal delivery: aOR: 1.37, 95 CI: 0.99–1.88; cesarean delivery: aOR: 1.39, 95% CI: 0.95–2.04	Maternal age, maternal race and ethnicity, maternal BMI, parity, GBS colonization status, chorioamnionitis, maternal asthma, maternal allergy, proportion of residents with less than high school education, median household income quartile, infant’s sex, birth weight-for-gestation z score, neonatal antibiotics, and breastfeeding at 3 mo
Kelderer, [36] 2022	Perspective birth cohort study, Sweden	1219	0–1.5	Parent reports through questionnaires	Parent reports	aOR: 0.92, 95% CI: 0.58–1.47	Infant sex, maternal and paternal history of allergic disease, exposure to pets (cats and dogs), and exposure to farm animals
Lin, [44] 2022	Retrospective nested case–control study, Taiwan	21,816	2.6 ± 2.9 (mean ± SD)	Database records	Database records (ICD codes)	aOR: 1.05, 95% CI: 0.98–1.12	Maternal and child’s age, gender, cesarean delivery, gestational hypertension/preeclampsia, gestational diabetes mellitus, pre-term/post-term, postnatal antibiotic exposure, neonatal hyperbilirubinemia, respiratory distress syndrome, congenital anomalies of heart, Kawasaki disease, allergic rhinitis, asthma, intussusception, febrile convulsion, epilepsy, ichthyosis, and child’s infections
Hong, [34] 2022	Retrospective cohort study, China	2909	0–2	Medical records	Parent reports of doctor’s diagnosis through questionnaires	Vaginal delivery: aOR: 6.56, 95% CI: 4.3–10.0; cesarean delivery: aOR: 0.56, 95% CI: 0.27–1.16	Maternal age, maternal allergy history, parity, gestational age, birth weight, feeding status, maternal pre-pregnancy BMI, maternal GBS status, infant major anomalies, maternal neuropsychiatric diseases, smoking, pet ownership, and antibiotic use in first 72 h of life
Puisto, [45] 2023	Prospective nested case–control, Finland	433	0–2	Prospectively documented study records	Clinically diagnosed by study physicians during control visits	aOR: 2.21, 95% CI: 1.20–4.10	Child’s sex, presence of older siblings, smoking during pregnancy, and breastfeeding duration
Vance, [43] 2023	Retrospective cohort, United States	9094	0–22	Parent reports through questionnaires	Doctor-diagnosed eczema reported by parents	aOR: 1.30, 95% CI: 1.09–1.57	Maternal age, body mass index of mother, smoking status during pregnancy, race, child’s birth weight, and parents’ history of food allergies, eczema, and asthma
Chang, [29] 2023	Retrospective cohort study, Taiwan	1,288,343	0.81 ± 1.1 (mean ± SD)	Database records	Database records (ICD codes)	aHR: 1.04, 95% CI: 1.03–1.05	Childbirth year, child’s sex, gestational age, birth weight, Apgar score, maternal age, urbanization, insurance property, mode of delivery, type of pregnancy, maternal acetaminophen use during pregnancy, maternal comorbidities, maternal atopic disorders, gestational infections, paternal age, and frequency of outpatient clinic visits during pregnancy
El-Heis, [32] 2023	Prospective cohort, United Kingdom	3158	0–1	Parent reports by interviews	Assessed by research nurses	Pooled aOR: 1.04, 95% CI: 0.92–1.18	Maternal BMI, parity, breastfeeding duration, and infant sex
Okoshi, [41] 2023	Prospective birth cohort study, Japan	78,678	0–3	Parent reports by interviews + medical records	Parent reports of doctor’s diagnosis through questionnaires	Doctor’s diagnosis aOR: 1.02, 95% CI: 0.97–1.08	Maternal age at delivery, parity, marital status, pre-pregnancy body mass index, pre-existing hypertension, pre-existing diabetes, maternal history of allergies, antipyretic or analgesic use during pregnancy, maternal education, household income, complication of pregnancy or delivery, morning sickness, weight gain during pregnancy, urinary cotinine concentration during pregnancy, alcohol consumption during pregnancy, sex of infant, premature birth, birth weight, breast-feeding, and pet ownership.
Stefanaki, [20] 2023	Prospective cohort study, Greece	236	0–1.5	Parent reports through questionnaires during interviews	Parent reports of doctor’s diagnosis through telephone interview	Pooled aOR: 2.03, 95% CI: 0.19–21.89	Maternal age, BMI, type of birth, smoking, maternal atopy, number of children, pregnancy paracetamol use, and type of yogurt consumed during pregnancy
Fuxench, [19] 2023	Prospective cohort study, United Kingdom	1,023,140	3.2 ± 4.6 (mean ± SD)	Database records (medical prescriptions)	Database records (diagnostic and prescription codes)	aHR: 1.38, 95% CI: 1.36–1.39	Maternal AD, seasonal allergies, asthma, gender assigned at birth, Townsend score (deprivation index), and mother’s race/ethnicity

Abbreviations: aOR, adjusted odds ratio; aHR, adjusted hazard ratio; BMI, body mass index; GBS, Group B *Streptococcus*; SD, standard deviation; AD, atopic dermatitis; ICD, International Classification of Diseases; mo, months old.

**Table 2:** Methodological quality of included studies, assessed by Newcastle–Ottawa Scale.

Author (Year)	Country	Study Design	Selection/4	Comparability/2	Outcome or Exposure/3	Score
Mckeever, [38] 2002	United Kingdom	Cohort study	****	*	***	8
Jedrychowski, [35] 2006	Poland	Cohort study	****	**	*	7
Dom, [31] 2010	Belgium	Cohort study	****	**	**	8
Wohl, [21] 2015	United States	Cohort study	***	**	***	8
Hesla, [37] 2017	Sweden	Cohort study	***	*	**	6
Timm, [42] 2017	Denmark	Cohort study	****	*	*	6
Metzler, [39] 2019	Austria, Finland, France, Germany and Switzerland	Cohort study	****	**	**	8
Gao, [33] 2019	China	Cohort study	****	**	**	8
Mubanga, [40] 2021	Sweden	Cohort study	****	**	***	9
Dhudasia, [30] 2021	United States	Cohort study	****	*	**	7
Lin, [44] 2022	Taiwan	Case–control study	***	**	***	8
Kelderer, [36] 2022	Sweden	Cohort study	***	**	*	6
Hong, [34] 2022	China	Cohort study	****	*	**	7
Stefanaki, [20] 2023	Greece	Cohort study	****	*	**	7
Puisto, [45] 2023	Finland	Case–control study	****	**	**	8
Vance, [43] 2023	United States	Cohort study	**	*	**	5
Chang, [29] 2023	Taiwan	Cohort study	****	**	***	9
El-Heis, [32] 2023	United Kingdom	Cohort study	****	*	***	8
Okoshi, [41] 2023	Japan	Cohort study	****	**	**	8
Fuxench, [19] 2023	United Kingdom	Cohort study	****	*	***	8

* Stars indicate the quality score assigned to each applicable category. A higher number of stars reflects better quality.

**Table 3:** Subgroup analysis and univariate meta-regression analysis. Significant *p* values are highlighted in bold.

Subgroups	Studies, *n*	Effect Size (95% CI)		Heterogeneity		Univariate Meta- Regression	
				*I* ^2^	*p*	β (SE)	*p*
**OVERALL STUDIES REPORTING ORs**	12	1.12	1.03–1.21	58	**0.006**		
**Study type**							
Cohort study *	11	1.16	1.04–1.30	62%	**0.003**	NA	
Case–control study	1	1.05	0.98–1.12	NA	NA	−0.10 (0.13)	0.44
**Study design**							
Prospective *	10	1.13	1.01–1.26	58%	**0.01**	NA	
Retrospective	2	1.15	0.93–1.41	78%	**0.03**	0.004 (0.11)	0.97
**Study region**							
Asia *	3	1.04	0.95–1.14	62%	0.07	NA	
Europe	8	1.19	1.01–1.39	51%	0.05	0.08 (0.09)	0.35
North America	1	1.30	1.08–1.56	NA	NA	0.21 (0.14)	0.14
**Sample size**							
≤1000 *	5	2.04	1.50–2.78	0%	0.86	NA	
1001–10,000	4	1.12	0.97–1.30	35%	0.20	−0.61 (0.16)	**0.0002**
>10,000	3	1.04	1.00–1.08	0%	0.62	−0.67 (0.16)	**<0.001**
**Publication year**							
≤2010 *	2	1.91	1.26–2.90	0	0.66	NA	
2011–2020	4	1.47	0.96–2.26	71%	**0.02**	−0.50 (0.23)	**0.03**
>2020	6	1.05	1.00–1.11	27%	0.23	−0.59 (0.22)	**0.009**
**Exposure assessment**							
Database or medical records *	2	1.03	0.99–1.08	0%	0.51	NA	
Parent-reported or interview	10	1.24	1.07–1.45	58%	**0.01**	0.14 (0.09)	0.12
**Outcome assessment**							
Database records or medical diagnoses *	2	1.05	0.99–1.11	0%	0.89	NA	
Parent-reported or interview	10	1.21	1.06–1.39	66%	**0.002**	0.13 (0.10)	0.22
**Adjusted for maternal infections/paracetamol use or child’s infections/antibiotic use**							
No *	6	1.14	1.01–1.29	52%	0.06	NA	
yes	6	1.11	0.98–1.26	64%	**0.02**	−0.02 (0.09)	0.82
NOS < 8 *	6	1.25	1.01–1.54	58%	**0.04**	NA	
**NOS ≥ 8**	6	1.06	0.98–1.16	55%	0.05	−0.10 (0.09)	0.27
**Adjusted for parental allergic diseases**							
No *	4	1.07	0.99	1.17	0.1	NA	
Yes	8	1.28	1.03	1.59	0.006	0.11 (0.13)	0.41
**OVERALL STUDIES PRESENTED WITH HR**	4	1.12	0.96–1.31	100%	**<0.00001**		
**Trimester of exposure**							
First	2	1.06	1.00–1.13	95%	**<0.00001**	0.01 (0.07)	0.84
Second *	2	1.05	0.99–1.11	94%	**<0.0001**	NA	
Third	2	1.06	0.95–1.18	99%	**<0.00001**	0.008 (0.07)	0.90
**Type of antibiotic**							
Penicillin	2	1.18	0.94–1.49	100%	**<0.00001**	0.02 (0.15)	0.91
Sulfonamides	2	1.08	0.93–1.26	96%	**<0.00001**	−0.07 (0.15)	0.64
Cephalosporins	2	1.14	0.93–1.39	100%	**<0.00001**	−0.02 (0.15)	0.90
Macrolides *	2	1.16	1.01–1.34	99%	**<0.00001**	NA	
**No. of courses**							
1–2 *	2	1.07	1.01–1.13	96%	**<0.00001**	NA	
3–4	2	1.08	1.03–1.14	78%	**0.03**	0.01 (0.04)	0.74
≥5	2	1.15	1.01–1.32	90%	**0.002**	0.06 (0.05)	0.22

Abbreviations: CI, confidence interval; OR, odds ratio; HR, hazard ratio; SE, standard error; NA, not applicable; NOS, Newcastle–Ottawa Scale. * Reference variable in meta-regression.

**Table 4:** Grading of Recommendations, Assessment, Development, and Evaluations (GRADE) assessment of included studies.

No. of Studies	Design	Effect	Limitations	Inconsistency	Indirectness	Imprecision	Publication Bias	Other Considerations	Certainty
**Risk of childhood atopic dermatitis with prenatal antibiotic use, presented with OR**									
12	11 cohort studies, 1 case–control studies	OR: 1.12, 95% CI: 1.03, 1.21	Serious (−1); residual confounding in some studies	Serious (−1); *I*^2^ > 50% observed in pooled analyses, some studies showed no effect	Not serious	Not serious	Detected (−1)		Very low
**Risk of childhood atopic dermatitis with prenatal antibiotic use, presented with HR**									
4	4 cohort studies	HR: 1.12, 95% CI: 0.96–1.31	Not serious	Serious (−1); *I*^2^ = 100%, one study showed no effect even if other three studies showed the same direction, and CIs barely overlap	Not serious	Serious (−1); wide CI that crossed 1.0	Detected by visual inspection (−1), even if Egger’s test showed non-significant *p* value	Dose–response relationship (+ 1)	Very low
**Risk of childhood atopic dermatitis with intrapartum antibiotic use**									
4	3 cohort studies, 1 case–control study	OR: 1.74, 95% CI: 1.08–2.82	Serious (−1); residual confounding in some studies	Serious (−1); *I*^2^ = 93%, direction of effect was not consistent	Not serious	Serious (−1); wide CI that crossed 1.0	Detected by visual inspection (−1), even if Egger’s test showed non-significant *p* value		Very low

Abbreviations: HR, hazard ratio; OR, odds ratio; CI, confidence interval.

## Data Availability

The data that support the findings of this study are available from the published studies included in the review or the corresponding author upon reasonable request.

## Supplementary Materials

The following supporting information can be downloaded at https://www.mdpi.com/article/10.3390/children12070859/s1: Table S1. PRISMA 2020 checklist; Table S2. Strategies used to search PubMed, Embase, and Cochrane; Figure S1. Directed acyclic graph of the hypothetical model illustrating the association between maternal antibiotic use during pregnancy and childhood atopic dermatitis; Figure S2. Sensitivity analysis with one study removal method of the main meta-analysis including 12 studies reporting ORs; Figure S3. Sensitivity analysis with one study removal method of a separate analysis including 4 studies reporting HRs; Figure S4. Funnel plot of the main meta-analysis of 12 studies; Figure S5. Sensitivity analysis with one study removal method regarding intrapartum antibiotic prophylaxis and risk of atopic dermatitis. Ref. [49] is cited in the supplementary materials.

## References

[1] Yew Y.W., Thyssen J.P., Silverberg J.I. (2019). A systematic review and meta-analysis of the regional and age-related differences in atopic dermatitis clinical characteristics. J. Am. Acad. Dermatol..

[2] Shin Y.H., Hwang J., Kwon R., Lee S.W., Kim M.S., Shin J.I., Yon D.K., GBD 2019 Allergic Disorders Collaborators (2023). Global, regional, and national burden of allergic disorders and their risk factors in 204 countries and territories, from 1990 to 2019: A systematic analysis for the Global Burden of Disease Study 2019. Allergy.

[3] Stander S. (2021). Atopic Dermatitis. N. Engl. J. Med..

[4] Langan S.M., Irvine A.D., Weidinger S. (2020). Atopic dermatitis. Lancet.

[5] Orwa S.A., Gudnadottir U., Boven A., Pauwels I., Versporten A., Vlieghe E., Brusselaers N. (2024). Global prevalence of antibiotic consumption during pregnancy: A systematic review and meta-analysis. J. Infect..

[6] Schilling A.L., Rody A., Bossung V. (2023). Antibiotic Use During Pregnancy and Childbirth: Prospective Observational Study on Prevalence, Indications, and Prescribing Patterns in a German Tertiary Center. Geburtshilfe Frauenheilkd..

[7] Russell N.J., Seale A.C., O’Sullivan C., Le Doare K., Heath P.T., Lawn J.E., Bartlett L., Cutland C., Gravett M., Ip M. (2017). Risk of Early-Onset Neonatal Group B Streptococcal Disease With Maternal Colonization Worldwide: Systematic Review and Meta-analyses. Clin. Infect. Dis..

[8] Russell N.J., Seale A.C., O’Driscoll M., O’Sullivan C., Bianchi-Jassir F., Gonzalez-Guarin J., Lawn J.E., Baker C.J., Bartlett L., Cutland C. (2017). Maternal Colonization With Group B Streptococcus and Serotype Distribution Worldwide: Systematic Review and Meta-analyses. Clin. Infect. Dis..

[9] Low J.M., Lee J.H., Foote H.P., Hornik C.P., Clark R.H., Greenberg R.G. (2024). Incidence of group B streptococcus early-onset sepsis in term neonates with second-line prophylaxis maternal intrapartum antibiotics: A multicenter retrospective study. Am. J. Obstet. Gynecol..

[10] Vuillermin P.J., Macia L., Nanan R., Tang M.L., Collier F., Brix S. (2017). The maternal microbiome during pregnancy and allergic disease in the offspring. Semin. Immunopathol..

[11] Sanidad K.Z., Zeng M.Y. (2020). Neonatal gut microbiome and immunity. Curr. Opin. Microbiol..

[12] Alhasan M.M., Holsken O., Duerr C., Helfrich S., Branzk N., Philipp A., Leitz D., Duerr J., Almousa Y., Barrientos G. (2023). Antibiotic use during pregnancy is linked to offspring gut microbial dysbiosis, barrier disruption, and altered immunity along the gut-lung axis. Eur. J. Immunol..

[13] Cait A., Wedel A., Arntz J.L., Duinkerken J., Datye S., Cait J., Alhasan M.M., Conrad M.L. (2022). Prenatal antibiotic exposure, asthma, and the atopic march: A systematic review and meta-analysis. Allergy.

[14] Huang F.Q., Lu C.Y., Wu S.P., Gong S.Z., Zhao Y. (2020). Maternal exposure to antibiotics increases the risk of infant eczema before one year of life: A meta-analysis of observational studies. World J. Pediatr..

[15] Tsakok T., McKeever T.M., Yeo L., Flohr C. (2013). Does early life exposure to antibiotics increase the risk of eczema? A systematic review. Br. J. Dermatol..

[16] Wan M., Yang X. (2023). Maternal exposure to antibiotics and risk of atopic dermatitis in childhood: A systematic review and meta-analysis. Front Pediatr.

[17] Zhong Y., Zhang Y., Wang Y., Huang R. (2021). Maternal antibiotic exposure during pregnancy and the risk of allergic diseases in childhood: A meta-analysis. Pediatr. Allergy Immunol..

[18] Page M.J., McKenzie J.E., Bossuyt P.M., Boutron I., Hoffmann T.C., Mulrow C.D., Shamseer L., Tetzlaff J.M., Akl E.A., Brennan S.E. (2021). The PRISMA 2020 statement: An updated guideline for reporting systematic reviews. Int. J. Surg..

[19] Fuxench Z.C., Mitra N., Del Pozo D., Hoffstad O., Shin D.B., Langan S.M., Petersen I., Bhate K., Margolis D.J. (2024). In utero or early-in-life exposure to antibiotics and the risk of childhood atopic dermatitis, a population-based cohort study. Br. J. Dermatol..

[20] Stefanaki E., Kalaitzidou I., Aristou M., Lakoumentas J., Galanakis E., Xepapadaki P. (2023). Prenatal antibiotic exposure increases the risk of infant atopic dermatitis: Data from a Greek cohort. Eur. Ann. Allergy Clin. Immunol..

[21] Wohl D.L., Curry W.J., Mauger D., Miller J., Tyrie K. (2015). Intrapartum antibiotics and childhood atopic dermatitis. J. Am. Board Fam. Med..

[22] Stang A. (2010). Critical evaluation of the Newcastle-Ottawa scale for the assessment of the quality of nonrandomized studies in meta-analyses. Eur. J. Epidemiol..

[23] Guyatt G.H., Oxman A.D., Vist G.E., Kunz R., Falck-Ytter Y., Alonso-Coello P., Schunemann H.J., Group G.W. (2008). GRADE: An emerging consensus on rating quality of evidence and strength of recommendations. BMJ.

[24] Meader N., King K., Llewellyn A., Norman G., Brown J., Rodgers M., Moe-Byrne T., Higgins J.P., Sowden A., Stewart G. (2014). A checklist designed to aid consistency and reproducibility of GRADE assessments: Development and pilot validation. Syst. Rev..

[25] George A., Stead T.S., Ganti L. (2020). What’s the Risk: Differentiating Risk Ratios, Odds Ratios, and Hazard Ratios?. Cureus.

[26] Dettori J.R., Norvell D.C., Chapman J.R. (2022). Fixed-Effect vs Random-Effects Models for Meta-Analysis: 3 Points to Consider. Glob. Spine. J..

[27] Ruppar T. (2020). Meta-analysis: How to quantify and explain heterogeneity?. Eur. J. Cardiovasc. Nurs..

[28] Sedgwick P. (2015). Meta-analysis: What is heterogeneity?. BMJ.

[29] Chang Y.C., Wu M.C., Wu H.J., Liao P.L., Wei J.C. (2023). Prenatal and early-life antibiotic exposure and the risk of atopic dermatitis in children: A nationwide population-based cohort study. Pediatr. Allergy Immunol..

[30] Dhudasia M.B., Spergel J.M., Puopolo K.M., Koebnick C., Bryan M., Grundmeier R.W., Gerber J.S., Lorch S.A., Quarshie W.O., Zaoutis T. (2021). Intrapartum Group B Streptococcal Prophylaxis and Childhood Allergic Disorders. Pediatrics.

[31] Dom S., Droste J.H., Sariachvili M.A., Hagendorens M.M., Oostveen E., Bridts C.H., Stevens W.J., Wieringa M.H., Weyler J.J. (2010). Pre- and post-natal exposure to antibiotics and the development of eczema, recurrent wheezing and atopic sensitization in children up to the age of 4 years. Clin. Exp. Allergy.

[32] El-Heis S., Crozier S.R., Harvey N.C., Healy E., Godfrey K.M. (2023). Early life exposure to antibiotics and laxatives in relation to infantile atopic eczema. Pediatr. Allergy Immunol..

[33] Gao X., Yan Y., Zeng G., Sha T., Liu S., He Q., Chen C., Li L., Xiang S., Li H. (2019). Influence of prenatal and early-life exposures on food allergy and eczema in infancy: A birth cohort study. BMC Pediatr..

[34] Hong Z., Jing R., Hui L., Kang X., Chunmei Z., Yang W., Baojian Z., Xin D., Xiaoping Y. (2022). A cohort study of intrapartum group B streptococcus prophylaxis on atopic dermatitis in 2-year-old children. BMC Pediatr..

[35] Jedrychowski W., Galas A., Whyatt R., Perera F. (2006). The prenatal use of antibiotics and the development of allergic disease in one year old infants. A preliminary study. Int. J. Occup. Med. Environ. Health.

[36] Kelderer F., Mogren I., Eriksson C., Silfverdal S.A., Domellof M., West C.E. (2022). Associations between pre- and postnatal antibiotic exposures and early allergic outcomes: A population-based birth cohort study. Pediatr. Allergy Immunol..

[37] Hesla H.M., Stenius F., Jarnbert-Pettersson H., Alm J. (2017). Allergy-related disease in relation to early life exposures-the ALADDIN birth cohort. J. Allergy Clin. Immunol..

[38] McKeever T.M., Lewis S.A., Smith C., Hubbard R. (2002). The importance of prenatal exposures on the development of allergic disease: A birth cohort study using the West Midlands General Practice Database. Am. J. Respir. Crit. Care Med..

[39] Metzler S., Frei R., Schmausser-Hechfellner E., von Mutius E., Pekkanen J., Karvonen A.M., Kirjavainen P.V., Dalphin J.C., Divaret-Chauveau A., Riedler J. (2019). Association between antibiotic treatment during pregnancy and infancy and the development of allergic diseases. Pediatr. Allergy Immunol..

[40] Mubanga M., Lundholm C., D’Onofrio B.M., Stratmann M., Hedman A., Almqvist C. (2021). Association of Early Life Exposure to Antibiotics With Risk of Atopic Dermatitis in Sweden. JAMA Netw. Open.

[41] Okoshi K., Sakurai K., Yamamoto M., Mori C., Japan E., Children’s Study g. (2023). Maternal antibiotic exposure and childhood allergies: The Japan Environment and Children’s Study. J. Allergy Clin. Immunol. Glob..

[42] Timm S., Schlunssen V., Olsen J., Ramlau-Hansen C.H. (2017). Prenatal antibiotics and atopic dermatitis among 18-month-old children in the Danish National Birth Cohort. Clin. Exp. Allergy.

[43] Vance T.M., Li T., Cho E., Drucker A.M., Camargo C.A., Qureshi A.A. (2023). Prenatal antibiotic use and subsequent risk of atopic eczema. Br. J. Dermatol..

[44] Lin T.L., Fan Y.H., Chang Y.L., Ho H.J., Wu C.Y., Chen Y.J. (2022). Early-life infections in association with the development of atopic dermatitis in infancy and early childhood: A nationwide nested case-control study. J. Eur. Acad. Dermatol. Venereol..

[45] Puisto R., Turta O., Rautava S., Isolauri E. (2023). Early life exposures and development of allergic disease in infants with familial risk: Results from ongoing probiotic intervention trials. Acta Paediatr..

[46] Grech A., Collins C.E., Holmes A., Lal R., Duncanson K., Taylor R., Gordon A. (2021). Maternal exposures and the infant gut microbiome: A systematic review with meta-analysis. Gut Microbes.

[47] Dharmage S.C., Lodge C.J., Lowe A.J., Allen K.J. (2015). Antibiotics and risk of asthma: A debate that is set to continue. Clin. Exp. Allergy.

[48] Protzko J., Schooler J.W. (2017). Decline effects: Types, mechanisms, and personal reflections. Psychological Science Under Scrutiny: Recent Challenges and Proposed Solutions.

[49] Page M.J., McKenzie J.E., Bossuyt P.M., Boutron I., Hoffmann T.C., Mulrow C.D. (2021). The PRISMA 2020 statement: An updated guideline for reporting systematic reviews. BMJ.

